# Constitutive or Inducible Protective Mechanisms against UV-B Radiation in the Brown Alga *Fucus vesiculosus*? A Study of Gene Expression and Phlorotannin Content Responses

**DOI:** 10.1371/journal.pone.0128003

**Published:** 2015-06-01

**Authors:** Emeline Creis, Ludovic Delage, Sophie Charton, Sophie Goulitquer, Catherine Leblanc, Philippe Potin, Erwan Ar Gall

**Affiliations:** 1 Sorbonne Université, UPMC Univ Paris 06, CNRS, UMR 8227, Integrative Biology of Marine Models, Station Biologique de Roscoff, CS 90074, F-29688, Roscoff cedex, France; 2 Centre de Ressources de Biologie Marine, MetaboMer Mass Spectrometry Core Facility, CNRS FR2424, Station Biologique de Roscoff, 29688, Roscoff cedex, Brittany, France; 3 Université Européenne de Bretagne, Université de Bretagne Occidentale, Laboratoire des Sciences de l’Environnement Marin, Unité Mixte de Recherche, Centre National de la Recherche Scientifique 6539, Institut Européen d’Etudes Marines-IUEM, 29280, Plouzané, Brittany, France; University of New South Wales, AUSTRALIA

## Abstract

A role as UV sunscreens has been suggested for phlorotannins, the phenolic compounds that accumulate in brown algae in response to a number of external stimuli and take part in cell wall structure. After exposure of the intertidal brown alga *Fucus vesiculosus* to artificial UV-B radiation, we examined its physiological responses by following the transcript level of the *pksIII* gene encoding a phloroglucinol synthase, likely to be involved in the first step of phlorotannins biosynthesis. We also monitored the expression of three targeted genes, encoding a heat shock protein (*hsp70*), which is involved in global stress responses, an aryl sulfotransferase (*ast*), which could be involved in the sulfation of phlorotannins, and a vanadium bromoperoxidase (*vbpo*), which can potentially participate in the scavenging of Reactive Oxygen Species (ROS) and in the cross-linking and condensation of phlorotannins. We investigated whether transcriptional regulation of these genes is correlated with an induction of phlorotannin accumulation by establishing metabolite profiling of purified fractions of low molecular weight phlorotannins. Our findings demonstrated that a high dose of UV-B radiation induced a significant overexpression of *hsp70* after 12 and 24 hours following the exposure to the UV-B treatment, compared to control treatment. The physiological performance of algae quantified by the photosynthetic efficiency (Fv/Fm) was slightly reduced. However UV-B treatment did not induce the accumulation of soluble phlorotannins in *F*. *vesiculosus* during the kinetics of four weeks, a result that may be related to the lack of induction of the *pksIII* gene expression. Taken together these results suggest a constitutive accumulation of phlorotannins occurring during the development of *F*.*vesiculosus*, rather than inducible processes. Gene expression studies and phlorotannin profiling provide here complementary approaches to global quantifications currently used in studies of phenolic compounds in brown algae.

## Introduction

The marine environment is highly contrasted from tidal zone to deeper waters. Based on tidal influence, ecological factors shape seaweed communities. The intertidal zone of cold and temperate rocky shores exposed to regular and extreme changes in abiotic conditions [1] is largely colonized by brown algae (mainly Fucales), which are especially experience to large variations of solar radiation, desiccation and osmotic stress during low tide. To resist and survive in this extreme environment, brown algae have acquired specific physiological traits during their independent evolution from the other major eukaryotic groups. As members of the Phylum Stramenopiles, brown algae have diverged over a billion years ago from land plants, red and green algae (Archaeplastida) and also from fungi and animals (Opisthokonts) [2]. This independent evolution has given way to many novel features with regard to their metabolism and cell biology.

One of these metabolic originalities is the synthesis of phenolic compounds which are specific to Phaeophyceae, namely phlorotannins. These metabolites are synthesized from the acetate-malonate pathway. The monomer synthesis consists in the condensation of malonyl-CoA units by a Polyketide Synthase of type III (PKS III) [3] leading to the formation of phloroglucinol (1,3,5-trihydroxybenzene) units. However further steps in the biosynthesis leading to the oligomerization of phloroglucinol units and condensation of high molecular weight phlorotannins still remain un-elucidated. Numerous studies have suggested that vanadium-dependent haloperoxidases may be involved in phlorotannin oxidative condensation [4][5][6] and probably play a major role in the processes leading to reactive oxygen species (ROS) detoxification [7]. The expert annotation of the *Ectocarpus* genome has also provided other candidates for phlorotannin biosynthesis such as arylsulfotransferases [8], which could be involved in the water solubility and stabilization of phenolic compounds [9]. Similarly to land plant polyphenols, the phlorotannins are likely to possess important ecological roles. Indeed phlorotannin contents in Phaeophyceae exhibit plasticity in their responses to a large variety of environmental factors, such as light, and nutrient availability, ultraviolet radiation and the intensity of grazing [10],[11],[12].

These metabolites occur under different forms in algal cells, soluble in cellular compartments like physodes [13][14] and in extracellular exudates [15], or insoluble cross-linked into the cell walls [16]. This apparent diversity of location, chemical speciation and functions of phlorotannins has currently been poorly explored. Therefore, even though global estimations of soluble phenol pools have been provided up to date [17], precise quantification of total phlorotannin contents in these different compartments still remains a challenge.

In this present study, we focused on the potential effect of UV-B on synthesis of phorotannins. Impacts of UV-B can be multiple [18], from DNA damage-inhibition of DNA replication or mutations-, to photosynthetic apparatus impairment-decrease of CO2-fixation, production of (ROS)- [19][20]. During acclimation to UV radiation, a reduction in the degree of photo-inhibition is commonly observed in brown algae. Such an effect may be explained either by activation of the antioxidative response, or by the formation of UV-screening compounds [21] and an increase in the activity of repairing enzymes. Phlorotannins are able to absorb UV radiation, mainly UV-C and partly UV-B, with maxima at 195 nm and 265 nm [22][23][24] making them good candidates for UV protection. Similarly to tannins from land plants, phlorotannins expose a high antioxidant activities, essential for the scavenging of toxic ROS, such as superoxide anion radicals produced by harmful UV-B radiation. However, results of previous studies are contrasted regarding the role of phlorotannins in brown algal responses upon UV radiations [23], [24],[25],[26],[27].

In this context, our study has the aim to study the chronic exposition of UV-B on physiological performance of *F*.*vesiculosus* by an original approach, combining targeted transcriptomic and quantification of phenolic compounds. The expression of target genes was monitored for a gene encoding the PKSIII enzyme responsible for the synthesis of phloroglucinol, the precursor of phlorotannins pathway, and for three other genes potentially involved in stress responses and phlorotannin modifications. The quantification of soluble phlorotannin content was completed with metabolite profiling of purified fractions of low molecular weight.

## Material and Methods

### Ethics Statement

The scientific research and collecting permits were obtained for the described field studies from the Maritime Affairs Authorities at DDTM29 (Department of Finistère, Brittany, France).

### Algal material and experimental design

*Fucus vesiculosus* was collected in October 2011 in the Bay of Brest near St Anne du Portzic (48.361591°N,-4.554375°W) at Plouzané (Brittany, France), thoroughly washed with filtered seawater and assigned in 40 liters tanks with a permanent renewal of both seawater and bubbled air. Three tanks were used as control with a permanent white light intensity of 70 μmoles.m^-2^.s^-2^ (Philips Master TL-D 36W/840) and three tanks were used for UV-B treatment with the same white light intensity plus an additional UV-B illumination of 4,9 W.m^-2^ UV-B and 0.09 W.m^-2^ UV-A (UV-B Q-Panel 313 nm). Each treatment was isolated from each other with dark sheets. The kinetic study lasted four weeks with 13 successive sampling points: after 0, 1, 3, 6, 12, 24, 36, 48, 72 hours, and 1, 2, 3, 4 weeks. The sampling consisted in the collection of one thallus in each tank, so three thalli under control treatment and three thalli under UV-B treatment.

### Fluorescence measurements and sampling

Photosynthetic efficiency was measured to determine the maximum quantum yield of PS II (*F*v/*F*m) by pulse amplitude modulated (PAM) fluorescence as Junior PAM Walz (Germany), with optical fiber and optical head as measuring units. Thalli were dark-adapted for 5 min prior to three independent measurements. After fluorescence assessment, total fresh tissues were cut off from three thalli of each condition, then frozen in liquid nitrogen and stored at -80°C before freeze-drying.

### Ribonucleic acid (RNA) extraction

The RNA extraction protocol was adapted from Apt & Grossman (1993)[28], Apt et *al*. (1995)[29] and Pearson et *al*. (2006)[30]. 50 mg dry weight (DW) of freeze-dried tissue were grounded for 5 min at 6500 rpm at room temperature using a mixer-mill (Precellys 24, Bertin Technologies) in 2 mL Eppendorf tubes with ceramic beads supplied. Extraction buffer consisted of Tris-EDTA (100 mM Tris, 50 mM EDTA, pH 7.5, with 1.5 M NaCl and 2% CTAB). Immediately prior to extraction, 500 mM of DTT was added as antioxidant from a 1 M stock dissolved in water. Extraction buffer was added to the tissue (1.5 mL per 50 mg dry weight) and the suspension was mixed vigorously by vortexing. After 15 min of extraction on ice, the mixture was centrifuged at 10000 g for 20 min at 4°C. The upper aqueous phase was transferred to a new tube and 1 volume of chloroform:isoamyl alcohol (24:1 v/v) was added, vortexed vigorously and centrifuged at 10000 g for 20 min at 4°C. The upper aqueous phase was transferred to a new tube and 0.2 volume of absolute ethanol was added gently and mixed by rocking the tube. Ethanol addition resulted in the precipitation of polysaccharides [31]. A second chloroform extraction was then carried out under the same conditions and the aqueous phase was carefully removed. RNA was precipitated with 0.4 volumes of 12 M LiCl in the presence of 1% (v/v) **β**-mercaptoethanol as antioxidant. Precipitation was performed overnight at -20°C. The RNA was collected by centrifugation at 10000 g for 30 min at 4°C, the RNA pellet was dried up (10–20 min on ice) and then re-suspended in 500 μL RNase-free water. The RNA was extracted with an equal volume of phenol-chloroform:isoamylalcohol (24:1 v/v) (1:1) and centrifuged at 10000 g for 15 min at 4°C. The resulting pellet was washed by 1 volume of chloroform:isoamyl alcohol (24:1 v/v) in order to remove the phenol. RNA was re-precipitated with 2 volumes ethanol, 0.3 M sodium acetate and re-suspended in 20 μL of sterile H_2_0. The RNA was treated with RNAse-free DNAse-I according to the manufacturer’s instructions (Qiagen) to remove any contaminating desoxyribonucleic acid (DNA). The concentration of RNA was assessed using a NanoDrop 2000 spectrophotometer (ThermoScientific), and the quality was assessed on a 2% agarose gel by running the gel electrophoresis at 80–100 V.

### Quantitative Real-time PCR (qRT- PCR)

The reverse transcription (RT) was performed on 250 ng of total RNA, with 1 μL DT18 (100 μM). The reaction mixture was incubated at 70°C for 5 min. Following the incubation the master mixture, which containes 5 μL Improm II Buffer 5X (Promega), 4 μL MgCl_2_ (25 mM), 1 μL dNTP mix (10 mM), 0.5 μL RNAsine, 2.5 μL Nuclease free water and 1 μL of ImProm-II Reverse Transcriptase (Promega) was added. The reaction mixture was incubated at 25°C for 5 min, the extension was carried out at 42°C for 60 min, and the RT was inactivated by heating at 70°C for 15 min. Finally, the total complementary DNA (cDNA) was diluted at 1 ng/μL to perform the reverse transcription polymerase chain reaction (qRT-PCR) which was carried out using *ef1α* and *tua* as endogenous control on the LightCycler 480 multiwell plate 96, on a LightCycler 480 Real-Time PCR System (Roche Diagnostics, Mannheim, Germany) in three technical replicates. The final reaction volume was made to 10 μL, using 5 μL of the LightCycler 480 SYBR Green Master mix (Roche Diagnostics, Mannheim, Germany) with 2.5 μL of cDNA (1 ng.μL^-1^) or *F*. *vesiculosus* cesium chloride-purified genomic DNA (gDNA) for quantification, 0.5 μL of each primer (10 μM) (Table 1) and 1.5 μL water.

**Table 1:** Primers used for the qRT-PCR analysis on control and UV-B conditions.

Gene	Forward	Reverse	Amplicon Length (bp)	Tm °C	Accession number	References
*ef1*.*α*	TGCGTACAATCGCATTCG	CGAAACATGAAGGACAGTTGC	198	58	GH706096	EST (Pearson et al. 2010)
*tua*	GTCACACCGATGTAGAGGA	GGCTTCCAGACAATTACCC	96	58	GH702736	EST (Pearson et al. 2010)
*pks*	TTGCACGTATGTCTCTGTTGC	GCGCGAATAACCTGATGG	135	60	GH706741	EST (Pearson et al. 2010)
*vbpo*	CCAAGGCGTCGAGTCATATC	GCACTTACTGCAATCCAATGTAC	129	59	comp9395_c0_seq3	I.Kruse, personal comm.
*hsp70*	AGATCGAGGAGATTGACTAGATGG	CGACTTGCATCACACATATCG	161	60	GH704979	EST (Pearson et al. 2010)
*ast6*	GACCCTTCCCTGATCTTCC	CCAGATGCGGTCATTTCAC	83	59	GH702197	EST (Pearson et al. 2010)

The cycling program for PCR quantification was as follows: 5 min denaturation at 95°C, followed by 45 cycles of 95°C for 10 s, 60°C for 15 s, and 72°C for 15 s.

### Gene expression study

Primer pairs were designed using Primer3plus (http://primer3plus.com/cgi-bin/dev/primer3plus.cgi).

The design of a set of primers (Table 1) was based on expressed sequence tag (EST) clones from cDNA libraries from desiccated *F*. *vesiculosus* and *F*. *serratus* [32]. For the *pksIII* gene, the EST available from *F*. *vesiculosus* corresponded to the three prime untranslated region (3’UTR) [32] those function were not assigned. In order to identify this gene the 3’end part of the cDNA sequence was obtained using rapid amplification of cDNA ends (RACE) approaches. The 3’end sequence was cloned using the protocol developed by Scotto-Lavino et *al*.(2006) [33].

### Phlorotannin extraction and semi-purification

100 mg dry weight (DW) of freeze-dried tissue were grounded for 5 min at 6500 rpm at room temperature, using a mixer-mill (Precellys 24, Bertin Technologies) in 2 mL Eppendorf tubes with metal beads supplied. Extraction buffer consisted of methanol:water (80:20) at pH 4.3. Extraction was performed three times successively on the powder in dark at 40°C during 30 min with agitation in a thermomixer (Eppendorf). The extract was centrifuged 10 min at 10000 g and the supernatant was removed. Methanol was evaporated in a speed-vacuum concentrator miVac Duo Concentrator (miVac, Genevac Limited, Ipswitch, UK) at 40°C and the total extract was lyophilized and weighed.

In order to purify the total extract, the protocol developed by Steevensz et *al*. (2012) [34] was tested on 3 g of powder with 30 mL of methanol:water (80:20) at pH 4.3. After three consecutive extractions at 40°C with agitation, the methanol was removed with a Büchi Rotovapor R-114 with a B-480 water heater set at 35°C. Then the extract was defatted three to four times using dichloromethane (1:1 v/v) partitioning and fractionated using a C18 Sep-Pak cartridge (6 cc; 500 mg), which had been preconditioned with 12 mL methanol followed by 18 mL Milli Q (MQ) water. A sample (200–300 mg) of the freeze-dried aqueous fraction was dissolved in 200 mL MQ water for fractionation on the Dionex AutoTrace 280 SPE (Thermo Scientific) and eluted with 20 mL methanol. Samples were dried under N_2_, freeze-dried and weighed.

### Quantification of soluble phlorotannins

The quantification of total soluble phorotannins in the extracts was performed using the adapted Folin-Ciocalteu method [35] with phloroglucinol used as standard (Sigma). Each sample was re-suspended in 1 mL methanol:water (80:20) at pH 4.3 and diluted to reach a concentration of 1 mg.mL^-1^. Quantification was carried out using multiwell plates (Nunc UV-Star 96 wells), 20 μL of extract (1 mg.mL^-1^) were added to 40 μL of Na_2_CO_3_ 20%, 130 μL MQ water and 10 μL Folin-Ciocalteu reagent (Sigma). The reaction was incubated at 70°C during 10 min with a cover in a thermocycler and the absorbance of the solutions was then measured at 750 nm in multiwell plates on a Safire^2^Tecan Multi-detection Microplate reader.

### Ultrahigh-pressure liquid chromatography coupled to mass spectrometry conditions for profiling of purified phlorotannins

Ultrahigh-pressure liquid chromatography was performed in the same conditions described by Steevensz et *al*. (2012) [34] using an Ultimate 3000 (Dionex). Mass spectrometry analyses were performed on LTQ-Orbitrap Discovery (Thermo Scientific). Separations were achieved using a Waters UPLC BEH Amide 1.7 μm (2.1 x 100mm) column, 5 μL injections and a flow-rate of 400 μL.min^-1^. Mobile phase A was composed of 10.0 mM ammonium acetate adjusted to pH 9.0 with ammonium hydroxide and mobile phase B was acetonitrile. The gradient consisted of an initial hold at 5% mobile phase A for 1 min, followed by a linear gradient to 35% A in 16 min, followed by re-equilibration for 5 min at 5% A, for a total run time of 22 min. In the negative ion mode, the electrospray voltage was set to 3.42 kV, the capillary voltage to 45 V, and the tube lens offset to 130 V. The sheath and auxiliary gas flows (both nitrogen) were set to 5 arbitrary units (a.u.), and the drying gas temperature was set to 300°C. Mass spectra were recorded from 50 *m/z* up to 1000 *m/z* at a resolution of 30 000 (FWHM at *m/z* 400) acquired in the centroid mode.

Following their acquisition by Xcalibur software (Thermo Fisher Scientific), metabolomic fingerprints were deconvoluted to allow the conversion of the three-dimensional raw data (*m/z*, retention time, ion current) to time- and mass-aligned chromatographic peaks with associated peak areas. Massmatrix File Conversion was used to convert the original Xcalibur data files (*.raw) to a more exchangeable format (*.mzXML). Data processing was then performed using the open-source Workflow4metabolomics.org. CentWave was used for the peak picking. The interval of *m/z* value was set to 0.1, the signal to noise ratio threshold was set to 10, the group band-width was set to 10 and the minimum fraction was set to 0.75. Obiwarp was used for retention time correction with profstep set to 0.1.

### Statistical analyses

All data obtained under the different experiments and conditions were analyzed using one-way analysis of variance (one-way ANOVA p < 0.05). Mean comparisons were made using LSMEANS test with significant differences reported at p < 0.05. All statistical analyses were done using R version 3.0.1 [36] with R packages [37],[38].

## Results

### Effect of UV-B chronic exposure on the physiological fitness of *Fucus vesiculosus*

**Fig 1:**
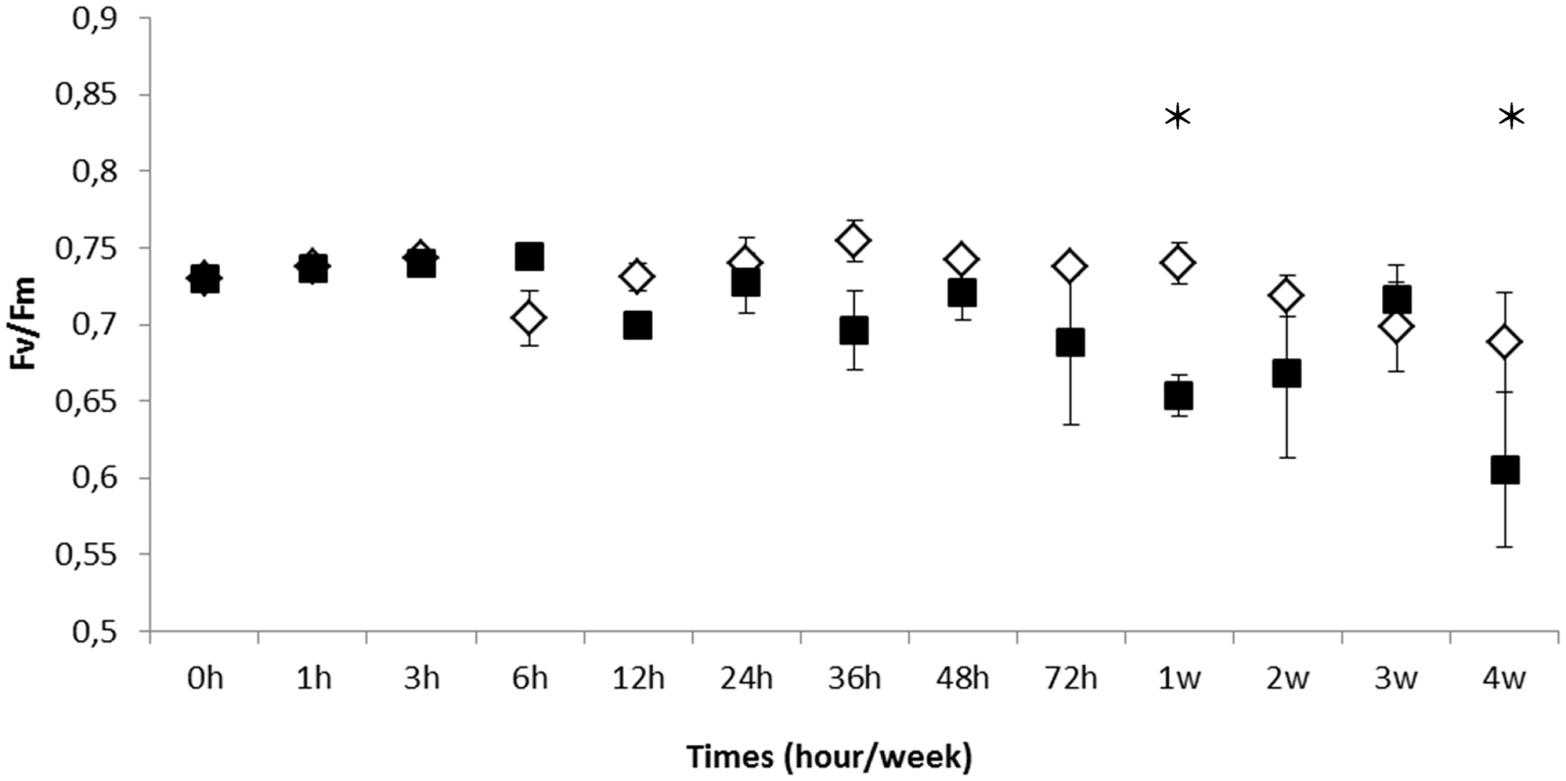
Photosynthesis efficiency of *Fucus vesiculosus* under chronic exposure to UV-B radiation during 4 weeks. The optimal quantum yield (Fv/Fm) of apical parts of *F*.*vesiculosus* thalli measured through imaging PAM fluorimetry during exposure to PAR (controls, white losanges) and UV-B condition (black squares).Values represent means of three independent replicates and bars represent the SE. Statistically significant differences (LSMEANS 0.05) between treatments are indicated by stars (*).

In our experimental conditions, the UV-B condition corresponded to a UV-B dose approximately two times more important than in full sunlight at noon in spring in Brittany [39]. This condition was likely to affect the alga physiological performance. In order to control this performance, changes in the optimal quantum yield (Fv/Fm) of apical parts of the *F*. *vesiculosus* thallus were measured after different times of exposure to UV-B radiation (Fig 1). During the first 3 days of UV-B exposure, no significant difference appeared compared to the control conditions. However, after one week and four weeks of chronic exposure, the maximum efficiency of PSII was significantly more affected by UV-B radiation in comparison with control conditions (ANOVA pvalue = 0.040; LSMEANS 0.05), with a slight reduction during the whole kinetics.

### Gene expression

**Fig 2:**
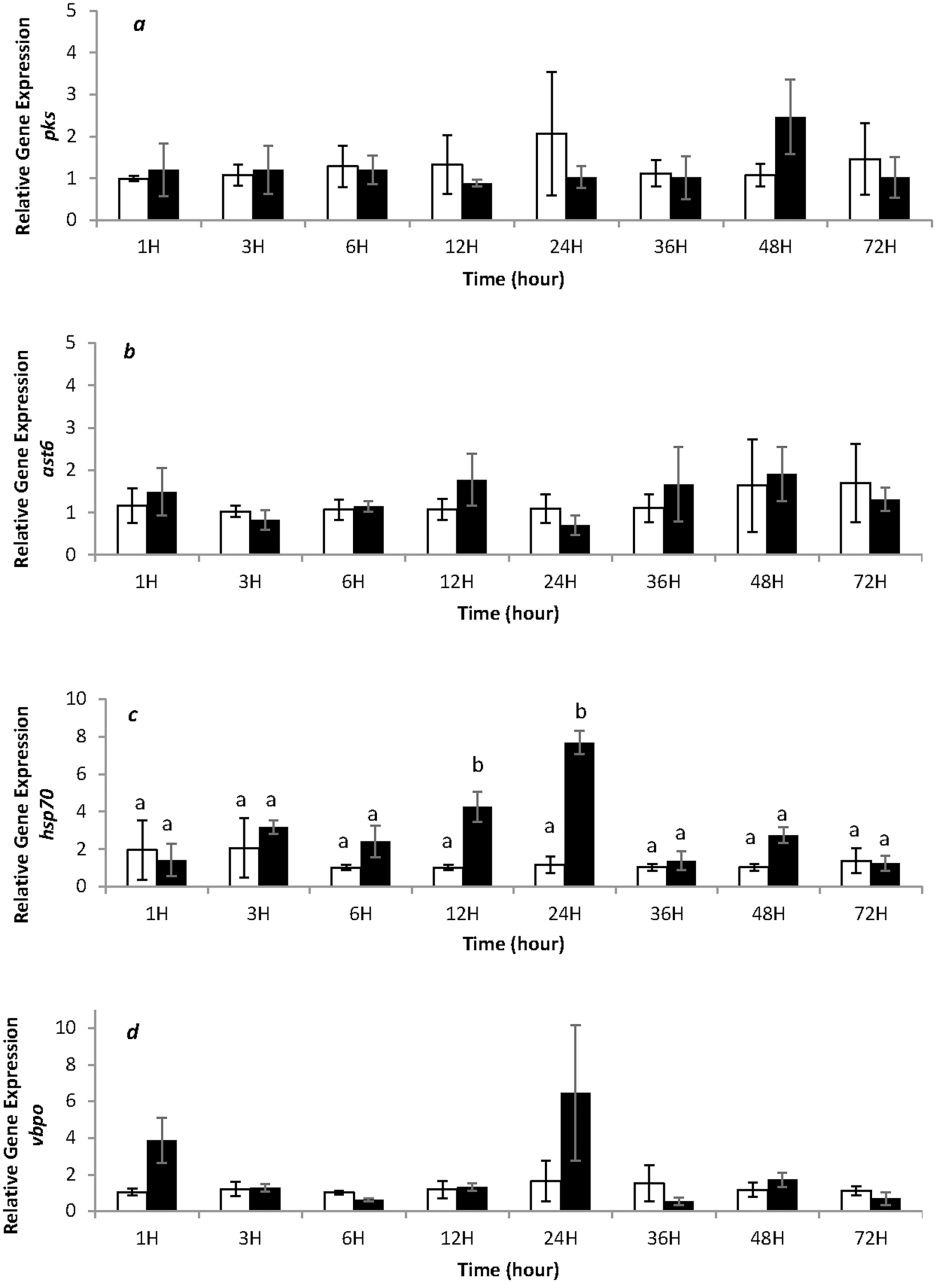
Relative gene expression in *Fucus vesiculosus* in controlled condition (white square) and exposed to UV-B (black square) during 72 hours are presented for *pksIII* (a), *ast6* (b), *hsp70* (c), and *vbpo* (d). The expression of a gene is normalized to the geometric mean of the expression of 2 reference genes (*ef1α*, *tua*) in the same algal sample and to the mean of its expression in the three control algae at each time point. Values represent means of three independent replicates and bars represent the SE. Letters indicate significant difference (LSMEANS 0.05).

A large number of ESTs available in the databases for *Fucus* spp. correspond to 3’UTR regions. Therefore, before designing primer sequences for qRT-PCR, we cloned a larger partial cDNA of a *pksIII* gene from *F*. *vesiculosus* in order to confirm the identity of the coding region of this sequence with other *pksIII* genes, including a biochemically characterized PKSIII enzyme, such as EsiPKS1 from the brown alga *Ectocarpus* [3]. The cDNA of *F*.*vesiculosus* cloned covered 67.5% of the total sequence of *EsiPKS1* with 1726 bp fragment. The corresponding amino acid sequence was 98.3% identical to the PKS1 from *Ectocarpus* (GenBank accession no: CBN76919) and exhibited significant similarities to algal and bacterial *pksIII* (Figs A–C in S1 File). The expression kinetics of *pksIII*, *ast6*, *hsp70* and *vbpo* genes was monitored by qRT-PCR (Fig 2). The relative expression of *pksIII* and *ast6* genes showed no significant regulation of these genes during UV-B exposure, expression levels ranged from 1 to 2.5 in fold-change (Fig 2a and 2b). However an effect of UV-B treatment was detected for *hsp70* gene (ANOVA, pvalue = 0.00376). In fact, *hsp70* gene (Fig 2c) was over-expressed four times at 12 h and eight times at 24 h in UV-B condition compared to control. Despite a relative expression more variable than *pksIII* and *ast6* gene, the relative expression of *vbpo* (Fig 2d) ranged from 0.6 to 6.47 did not show any significant difference between control and UV-B treatment due to a high inter-individual variability.

### Phenol contents

**Fig 3:**
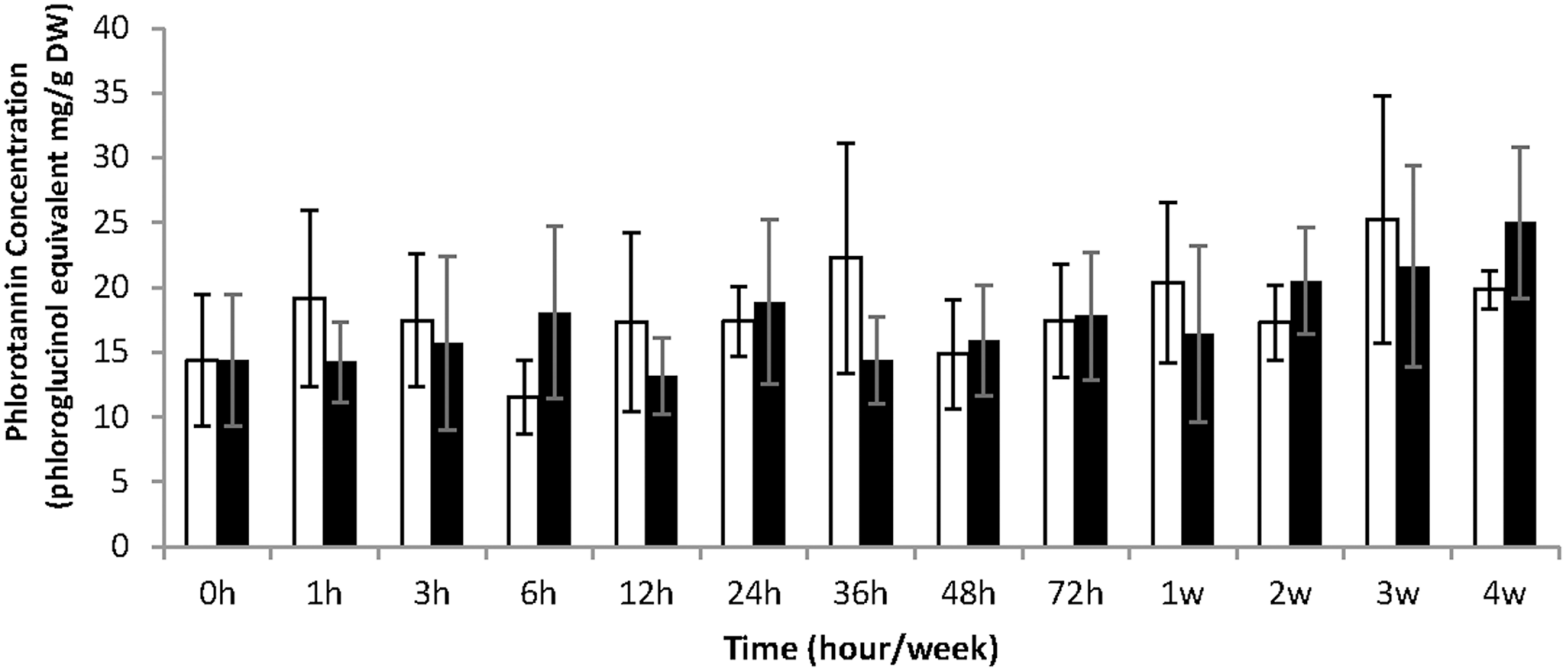
Quantification of total soluble phenol contents (mg equivalent phloroglucinol.g^-1^ DW) before purification in controlled condition (white square) and exposed to UV-B (black square). Values represent means of three independent replicates and bars represent the SD.

The total soluble phenol concentrations were measured by Folin-Ciocalteu method in methanolic extracts of the *Fucus vesiculosus* thallus, sampled after different times of exposure to UV-B radiation, and compared to those of control algae (Fig 3). The concentration of soluble phlorotannins ranged from 12 to 23 mg.g^-1^ DW, according to the phloroglucinol standard curve and the standard deviation (SD) calculated on three biological replicates, and showed a large variability between individuals. Indeed, the statistical analysis of the results revealed no significant difference between control and UV-B treatment (ANOVA, pvalue = 0.7381) and no effect of the kinetics (ANOVA, pvalue = 0.0696).

**Fig 4:**
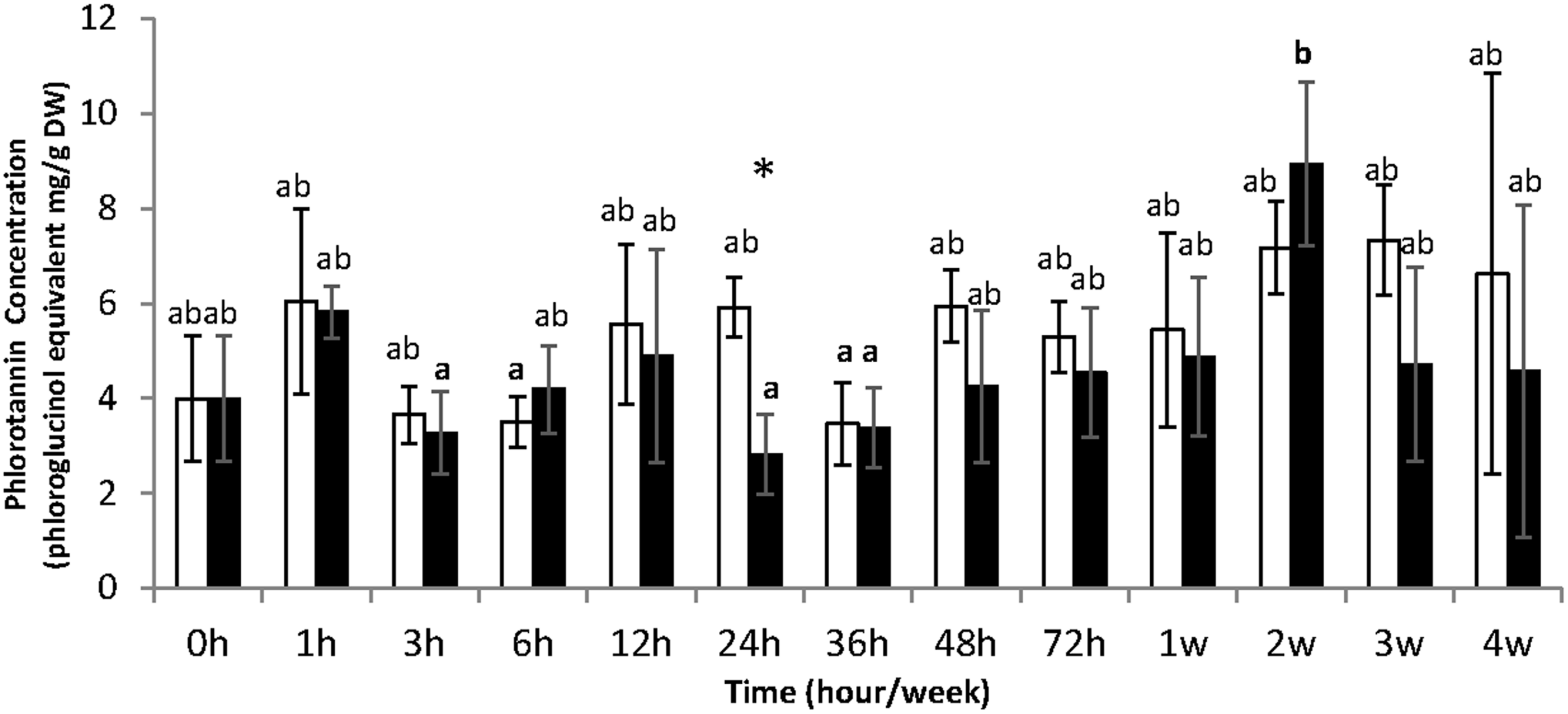
Quantification of soluble phenol contents in semi-purified phlorotannins fractions (mg equivalent phloroglucinol.g^-1^ DW) in controlled condition (white square) and exposed to UV-B (black square). Values represent means of three independent replicates and bars represent the SD. Mean with different letters indicate significant difference between all samples along the kinetic (LSMEANS 0.05). Stars (*) indicate significant difference between control and UV-B treatment (LSMEANS 0.05).

In semi-purified fractions (Fig 4), soluble phenol concentrations were in the range 3–8.9 mg.g^-1^ DW equivalent phloroglucinol, indicating a significant loss of compounds during the semi-purification process. The quantification of semi-purified fractions pointed out a significant effect of kinetics (ANOVA, pvalue = 0.001), with the lowest concentration in algae sampled after 24 hours of UV-B treatment (2.81 mg.g^-1^DW) and the most concentrated extract in those collected after 2 weeks of UV-B treatment (8.93 mg.g^-1^DW). A light but significant effect of UV-B treatment was also detected (ANOVA, pvalue < 0.1) and we observed a decrease of phenolic content from 5.9 mg.g^-1^DW equivalent phloroglucinol in control algae to 2.81 mg.g^-1^DW for treated algae with UV-B at 24 hours.

### Profiling of purified phlorotannins

**Fig 5:**
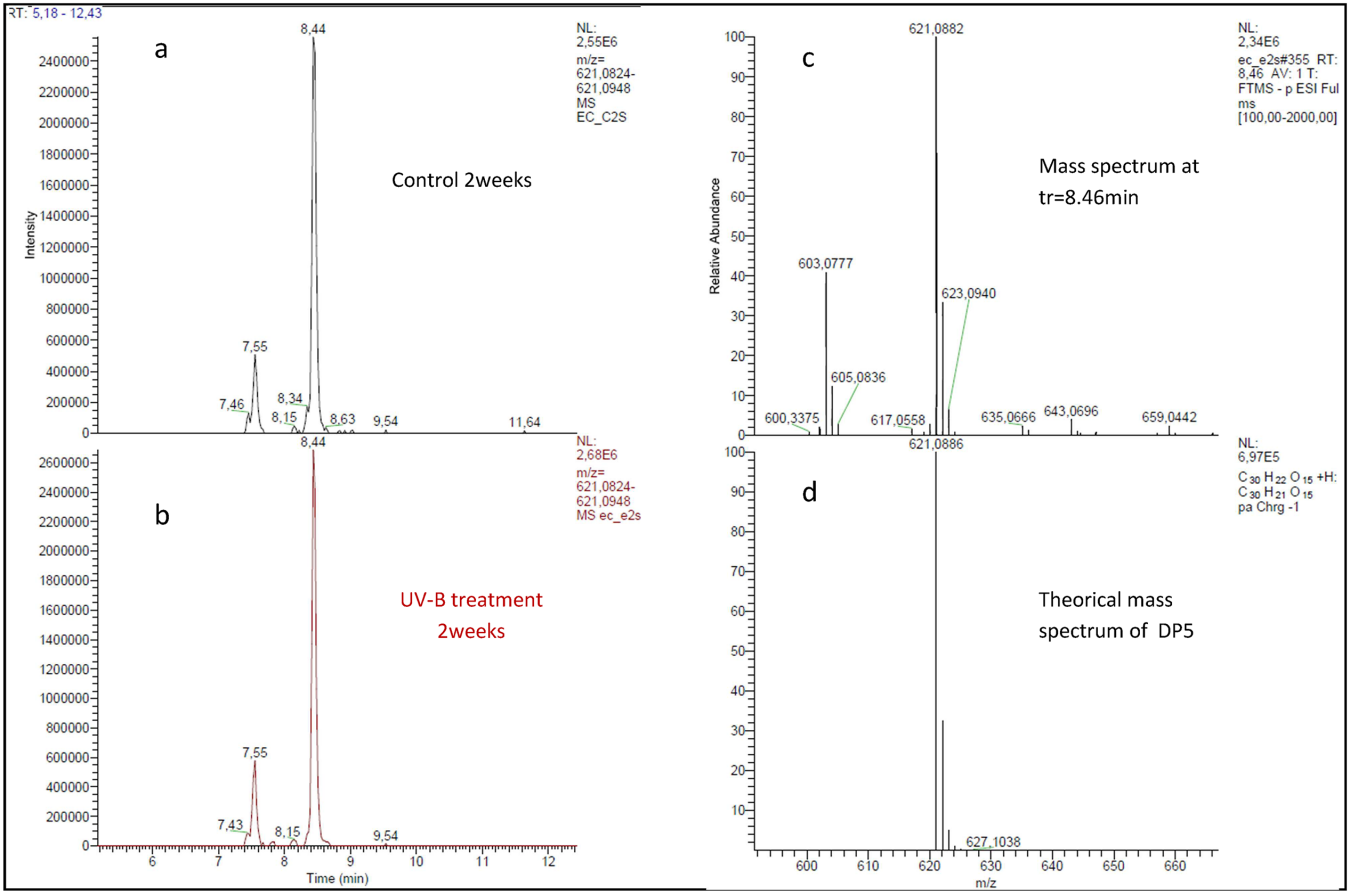
Ultra-HPLC-ESI-MS chromatogram of a precursor ion at *m/z* 621.0886 corresponding to a DP5 oligomer of phlorotannin in semi-purified fractions extracted from control (a) and UV-B treated algae (b) after 2 weeks. Mass spectrum from the chromatogram identified as DP5 (c). The theorical mass spectrum of DP5: C_30_H_21_O_5_ in the negative ion mode ([M-H]- = 621.0886 m/z) (d).

The U-HPLC-ESI-MS analyses of purified extracts evidenced several degrees of polymerization of phlorotannins from DP3 to DP7 (data not shown). However, after quantification of DP3 to DP7, no significant difference has been observed between control and UV-B treatment during the kinetic study (ANOVA pvalue = 0.84667). Fig 5 represents the chromatograms of an extraction of the precursor ion at *m/z* 621.0882 corresponding to a DP5 oligomer of phlorotannin in a control (Fig 5a) and UV-B treatment (Fig 5b) after two weeks. At least four isomers were detectable but no significant difference in their distribution and quantification upon UV treatment has been established compared to control treatment.

## Discussion

In order to cope with high levels of UV-B radiation, algae of the intertidal zone have developed strategies to survive and adapt in this fluctuating environment [40]. In terrestrial plants, both constitutive and inducible protection mechanisms have been reported [41]. UV-B radiations affect algae at different levels from degradation of the photosynthetic apparatus to DNA damage. However, the cumulative potential effects of this chronic stress on intertidal seaweeds at low tide and the inducibility of phlorotannin metabolism are poorly understood [24].

To address this question, we applied molecular and chemical analytical tools which could bring a major step forward in seaweed physiology [18].

### UV-B radiations upregulate the expression of *hsp70*, but do not regulate genes involved in phlorotannin metabolism

This study monitored the expression profile of targeted genes involved in the stress responses and in the metabolism of phlorotannins in brown algae. Our analysis using qRT-PCR has evidenced a significant effect of UV-B treatment on the expression level of *hsp70* at 12 h and 24 h, while no significant difference was detected in the expression of the three other genes. In our study the non-induction of the *pksIII* seems to be related with the non-induction of *vbpo* and *ast6* genes which encode proteins potentially implicated in modification of phlorotannins. However the presence of multigenic families in brown algae has been reported for the *vbpo* [7] and *ast* [42] that could reveal constitutive and inducible gene expression in these families.

A targeted approach was also recently attempted to evaluate the responses to thermal stress in *F*.*serratus* from four European populations along a large gradient of latitude [43]. This study has specifically measured the stress response to common-garden heat stress (20°C—36°C) and pointed a lack of correlation between the upregulation of *hsp70* and the decrease of photosynthetic performance possibly revealing a protective action of HSP70 proteins.

Other recent approaches on gene expression applied to ecophysiological studies in brown algae rather used global transcriptomics based on DNA microarrays or EST sequencing. In Fucales, the monitoring of the gene expression changes upon grazing in *F*. *vesiculosus* [44],[45] or desiccation stress in *Fucus* spp. [32] provided a large overview of gene categories that are regulated in response to these challenges. However, activation of specific pathways involved in both defense and desiccation tolerance remains to be investigated. Such a global transcriptomics was not yet attempted to specifically monitor gene expression in a brown alga in response to UV radiation.

### UV-B radiations slightly affect the photosynthetic performances, but do not promote phlorotannin accumulation in *Fucus vesiculosus*

The maximum capacity of the PSII seemed to be affected only after 1 week of chronic radiation, suggesting that photosynthetic efficiency of *F*. *vesiculosus* remained globally stable in culture, with a relative good tolerance of this species to UV-B radiation in opposition to the sensitivity of Laminariales [46]. The quantification of photosynthetic efficiency (Fv/Fm) gave values ranging from 0.6 to 0.75, which are comparable to results previously reported in fucoids [47]. In this context, it can be hypothesized that the alteration of photosynthetic performances might be buffered by the regulation of protein chaperones such as HSP70 and other mechanisms.

The high intensity (4.9 W.m^-2^) of UV-B also did not induce the accumulation of soluble phlorotannins in *F*. *vesiculosus* tissues after four weeks. Contents ranged from 12 to 23 mg.g^-1^ DW in the total methanolic extract, still comparable to levels found in others Fucales [48]. To complete this global quantification, we tested the hypothesis that the proportion of low molecular weight versus high molecular weight phlorotannins may shift upon UV exposure. Indeed the phlorotannin profiling can give more physiological information than the total phenol content [49]. We purified phlorotannins following a procedure involving a solid phase extraction before U-HPLC-MS analysis, recently developed by Steevensz et *al*.[34]. Phenol concentrations in semi-purified fractions were in the range 3–9 mg.g^-1^ DW equivalent phloroglucinol, indicating a significant loss of compounds during the semi-purification process. However, a significant effect of kinetics has been detected (Fig 4) that could indicate a significant variation in the extraction efficiency of semi-purified phlorotannins. The use of U-HPLC separation gave access to several degrees of polymerization (DP), from DP3 to DP7, with a good resolution but no significant difference in their distribution and quantification upon UV treatment has been established compared to control treatment (Fig 5).

Previous reports in brown algae suggested that the accumulation of soluble phlorotannins in response to UV stress were dependent of species, seasons and the development stage of thalli. In fact, a study in the Fucales *Ascophyllum nodosum* [23] has shown an increment of 30% of phlorotannin content after two weeks of exposure to UV-B radiation at 0.6 W.m^-2^. In contrast in embryos and juveniles of *Fucus gardneri* [24], no effect of UV-B has been detected on the phlorotannins content during a treatment of three weeks. Seasonal variations of phenol contents have been also demonstrated in *Fucus vesiculosus* [48], with the highest levels in spring like in the Laminariales *Lessonia nigrescens* [46], with significant increase of phlorotannin content during UV exposure in summer, when the development of sporophytes is optimal. Regarding these results, we can hypothesize that the induction of phlorotannin synthesis is linked to changes in the general metabolism such as increased photosynthesis. Other stimuli than UV-B, such as those provided by physical damage have previously shown a phlorotannin production over 20% in *Fucus distichus* in less than 2 weeks [50].

In the present study that combined biochemical and molecular approaches, the lack of accumulation of soluble phlorotannins over the kinetics is well related to the lack of over-expression of the gene encoding for PKSIII and others genes potentially related to phlorotannin modifications (*ast6*, *vbpo*), suggesting that this metabolism is not activated. However from our data, we cannot exclude a turn-over of some phenols [51] potentially insolubilized in the cell walls or secreted that would not require transcriptional activation of the *pksIII* gene, but only translational mechanisms or substrate availability for *de novo* synthesis. All available data point that the capacity of acclimatization of *F*. *vesiculosus* to drastic UV exposure would involve constitutive expression of the metabolism of phlorotannins, as already shown in terrestrial plants for UV-sunscreens, such as flavonoids [52].

In summary, this work highlights the adaptation of *Fucus vesiculosus* to elevated UV-B radiation in the intertidal zone. Taken together, the above results indicate that the stress is moderated and the effect of strong UV-B radiations is buffered by protective mechanisms. A constitutive accumulation of phlorotannins occurring during the development of *F*. *vesiculosus*, rather than inducible processes is likely essential for the acclimatization to excess of UV-B-radiations. Phenols are known to provide in these species efficient UV-sunscreens, [22, 23]. This adaptative constitutive mechanism prevents additional metabolic costs in response to variable environmental conditions of the intertidal zone.

Finally, the use of molecular tools to further investigate the regulation of phlorotannin biosynthesis appears particularly complementary of global quantifications and will be suitable to follow developmental changes in the phlorotannin synthesis or responses to various stresses in brown algae.

## Supporting Information

S1 File Fig A, Nucleotide alignment of the *Fucus vesiculosus* cDNA sequence with *Fucus* EST sequences. Fig B, Nucleotide alignment of the brown algal PKS III coding sequences. Fig C, Protein sequence alignment of the brown algal PKS III with a bacterial counterpart.
(DOCX)
