# Supplementary material for: Constitutive or Inducible Protective Mechanisms against UV-B Radiation in the Brown Alga Fucus vesiculosus? A Study of Gene Expression and Phlorotannin Content Responses
Source: PLoS One. 2015 Jun 1;10(6):e0128003. doi: 10.1371/journal.pone.0128003 (PMC4452539; doi:10.1371/journal.pone.0128003)
Supplement: S1 File — Fig A, Nucleotide alignment of the Fucus vesiculosus cDNA sequence with Fucus EST sequences. Fig B, Nucleotide alignment of the brown algal PKS III coding sequences. Fig C, Protein sequence alignment of the brown algal PKS III with a bacterial counterpart. (DOCX) [file pone.0128003.s001.docx]

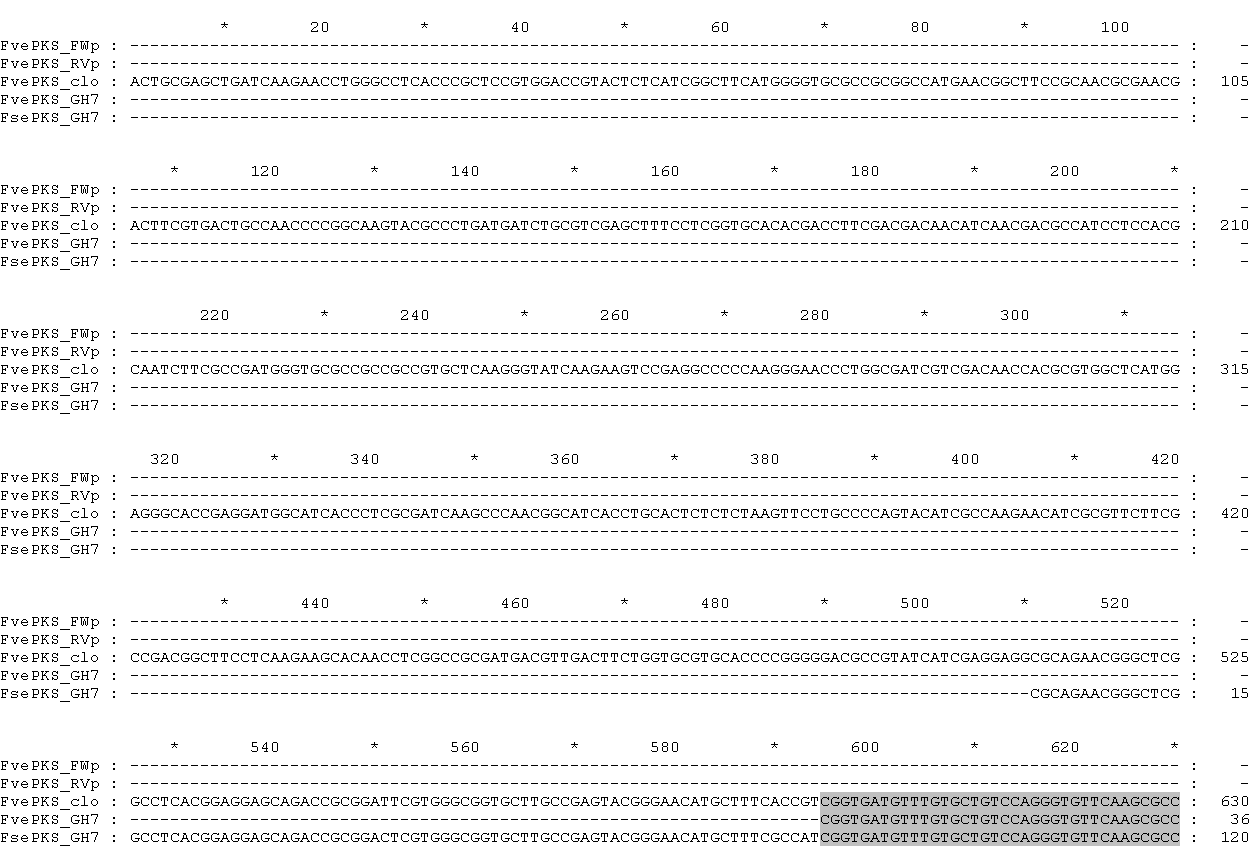


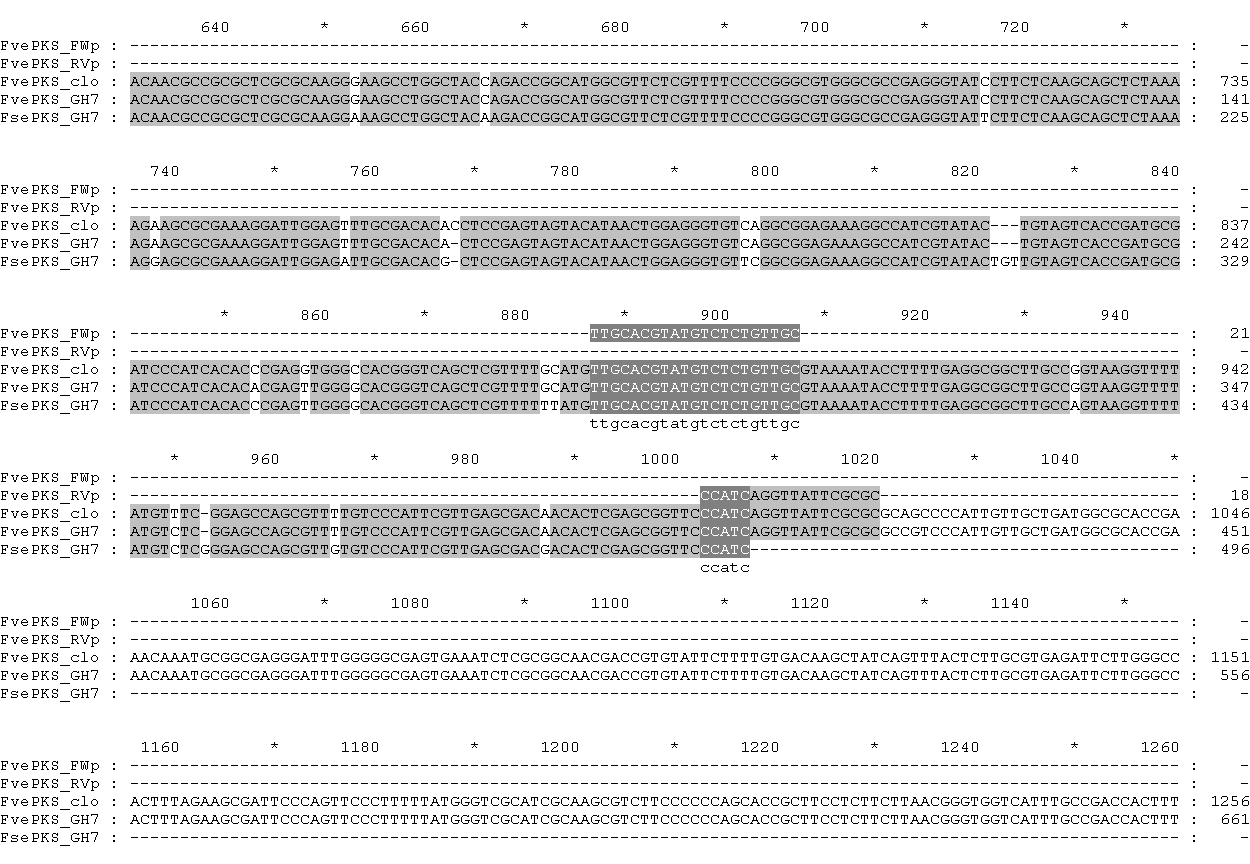


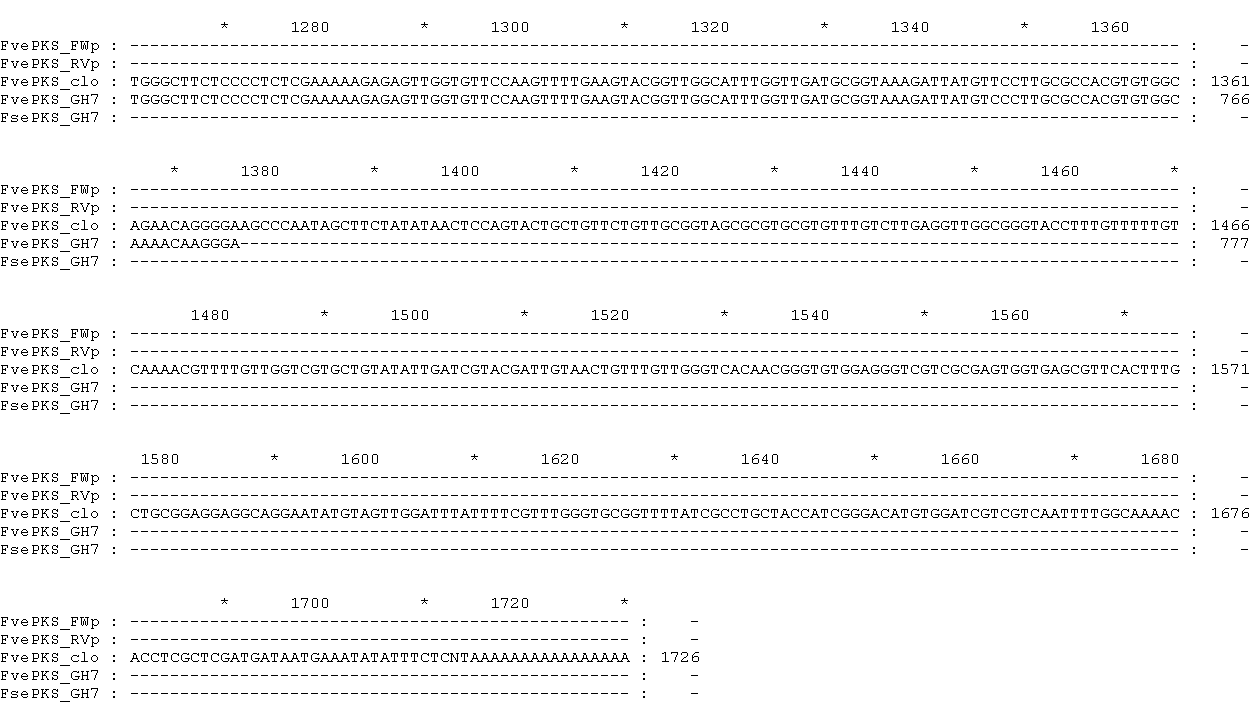


**S1 Fig.A: Nucleotide alignment of the brown algal PKS III coding sequences.**

**Nucleotide alignment of the *Fucus vesiculosus* cDNA sequence with Fucus EST sequences.**

qPCR forward primer (FvePKS_FWp), qPCR reverse primer (FvePKS_RVp), partial cDNA sequence cloned from *F. vesiculosus* (FvePKS_clo), *F. vesiculosus* EST of GH706741 GenBank accession (FvePKS_GH7), *F. serratus* EST of GH701018 GenBank accession (FsePKS_GH7). The positions sharing a conservation of 100, 80 and 60% are respectively shaded in black, dark grey and light grey.


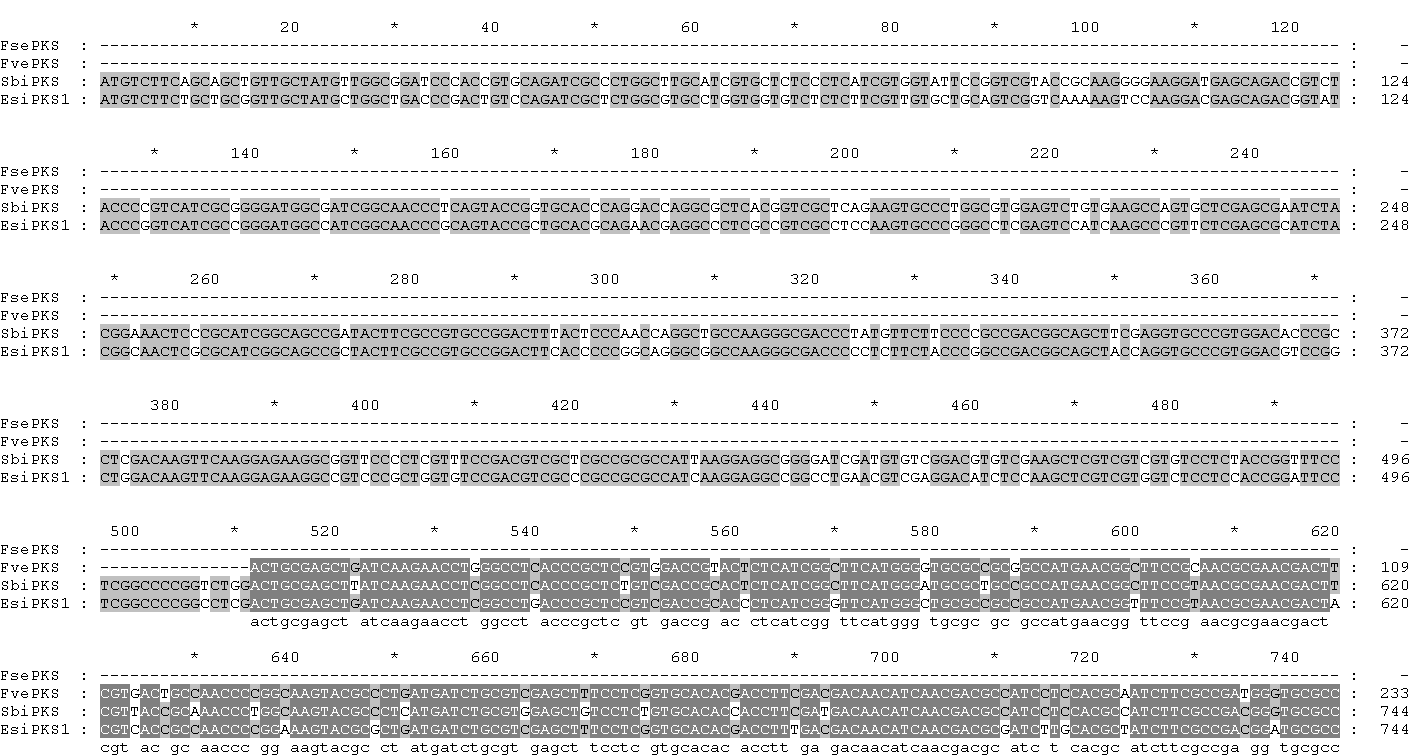


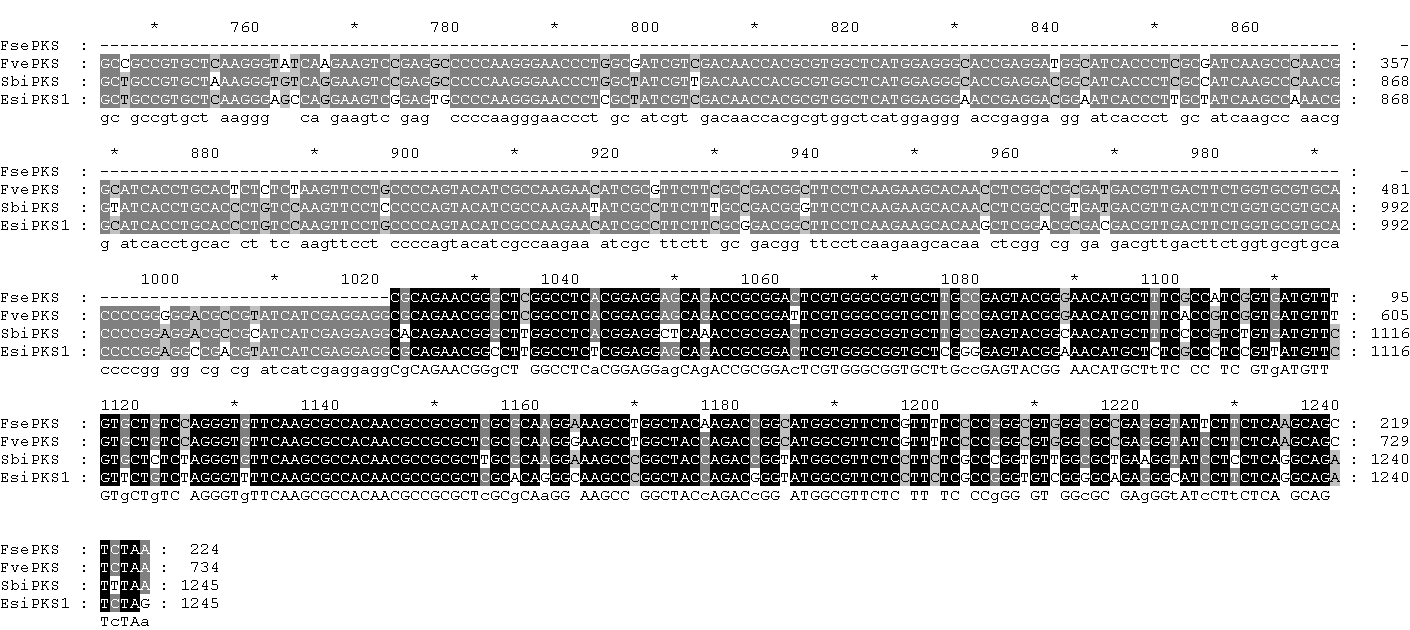


**S1 Fig. B: Nucleotide alignment of the brown algal PKS III coding sequences.**

*Fucus serratus* GH701018 (FsePKS), partial cDNA sequence cloned from *Fucus vesiculosus* (FvePKS), *Sargassum binderi* HM245964 (SbiPKS), *Ectocarpus siliculosus* Esi0024_0032 ORCAE Id (EsiPKS1). The positions sharing a conservation of 100, 75 and 50% are respectively shaded in black, dark grey and light grey.


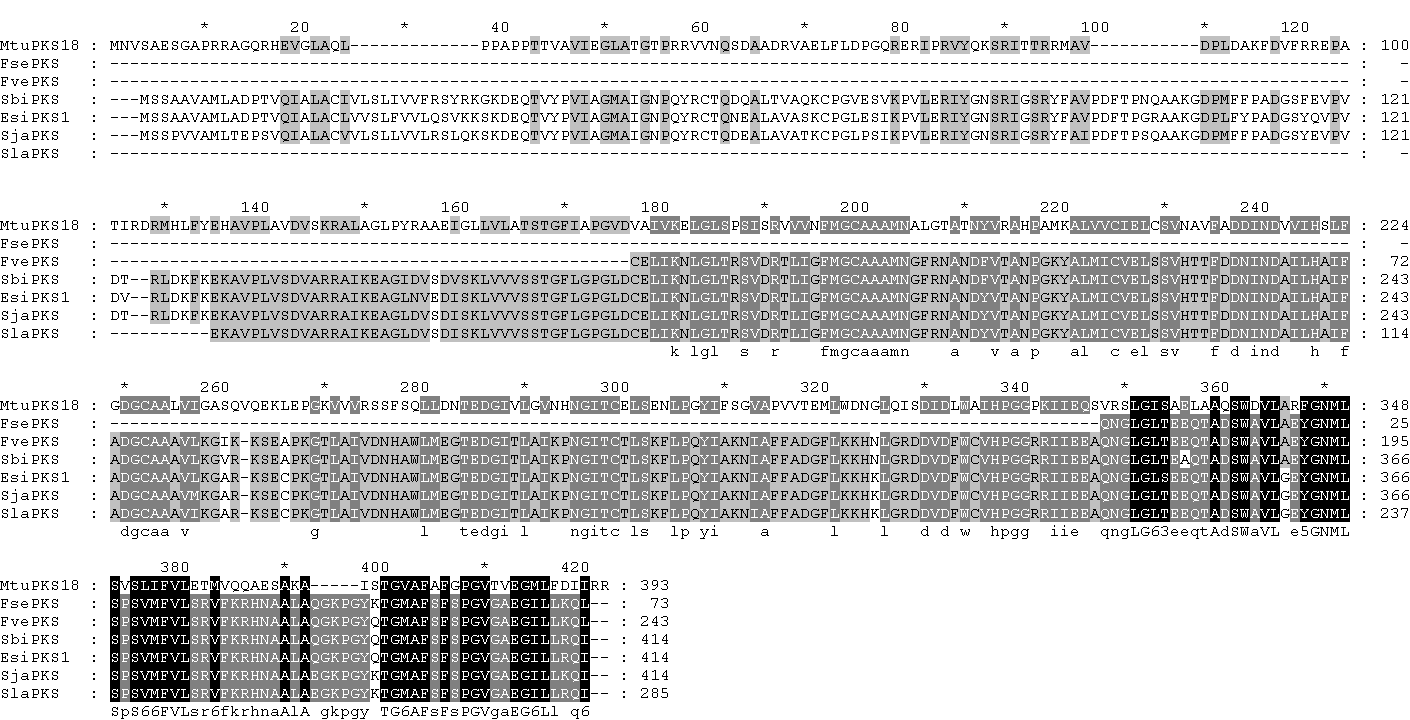


**S1 Fig.C: Protein sequence alignment of the brown algal PKS III with a bacterial counterpart.**

*Mycobacterium tuberculosis* P9WPF0 (MtuPKS18), translated sequence of *Fucus serratus* GH701018 (FsePKS), translated sequence of *Fucus vesiculosus* (FvePKS), *Sargassum binderi* ADK13089 (SbiPKS), *Ectocarpus siliculosus* Esi0024_0032 (EsiPKS1), translated sequence of *Saccharina japonica* contig_6991 (SjaPKS), translated sequence of *Saccharina latissima* contig_4304 (SjaPKS). The residues sharing 100, 80 and 60% identity are respectively shaded in black, dark grey and light grey.
